# Medication quality and quality of life in the elderly, a cohort study

**DOI:** 10.1186/1477-7525-9-95

**Published:** 2011-11-03

**Authors:** Inger Nordin Olsson, Rebecka Runnamo, Peter Engfeldt

**Affiliations:** 1 Family Medicine Research Centre, School of Health and Medical Sciences, Örebro University P.O. Box 1613, SE-701 16 Örebro, Sweden; 2 The National Board of Health and Welfare Regional Supervisory Unit Central P.O. Box 423, SE-701 48 Örebro, Sweden; 3 Faculty of Health Sciences, Linköping University, SE- 581 83 Linköping, Sweden

## Abstract

**Background:**

Modern drugs have made large contributions to better health and quality of life. Increasing proportions of negative side effects due to extensive pharmacological treatment are however observed especially among elderly patients who have multiple health problems. The aim of our study was to see if there is an association between medication quality and quality of life.

**Methods:**

150 patients discharged from hospital. Inclusion criteria were: living in ordinary homes, ≥ 75 years and ≥ 5 drugs. Home visits were performed to all, including prescription reviews and calculation of medication appropriateness index. The patients were divided into three groups depending on index score and followed for 12 months. The validated and recognized EQ-5D and EQ VAS instruments were used to assess quality of life.

**Results:**

A lower medication quality was associated with a lower quality of life. EQ-5D index was statistically significantly different (declining for each group) among the groups (p = 0.001 at study start, p = 0.001 at 6 months and p = 0.013 at 12 months) as was EQ VAS (p = 0.026 at study start, p = 0.003 at 6 months and p = 0.007 at 12 months).

**Conclusions:**

This study has shown the validity of the basic principle in prescribing: the more appropriate medication the better quality of life. Since drug quality is related to the patients' quality of life, there is immense reason to continuously evaluate every prescription and treatment. The evaluation and if possible deprescribing should be done as a process where both the patient and physician are involved.

## Background

The ageing process and becoming old is a complex phase encompassing many perspectives, for example loss of functions and decreasing autonomy, higher morbidity and need of care. With an ageing population the real challenge for the healthcare system is the increasing burden of chronic diseases and ongoing chronic medication [1]. Modern drugs have made great contributions to health and quality of life (QoL), though increasing proportions of negative side effects due to extensive pharmacological treatment are observed. Prescribing for older people demands specific knowledge [2,3]. Multi-medication or polypharmacy, defined as ≥ 5 drugs [4,5] is among the most obvious signs of risks in drug treatment, resulting in increased risks for inappropriate drug use and adverse drug reactions, followed by higher morbidity and hospitalization [6-9]. Polypharmacy also include risks of underutilization of each drug and underprescription of appropriate drugs [10-12] all possibly affecting QoL. Drug treatment can be either the facilitator which gives the opportunities, or the opposite, an intensifier of problems by occurrence of unacceptable side effects leading to decreased QoL.

Compared to other age groups there is a greater impact of health and functional ability on QoL in older ages [13,14]. If the goal of healthcare is both "to help people live longer and feel better" [15] there is a need for new outcome measures including QoL. In the area of medicine this demands a paradigm shift towards shared decision and incorporating the patient's preferences when the crucial factor is QoL [15]. The standardised and non-disease specific EQ-5D instrument [16] is used to assess the patient's health related QoL. Together with their self-rated QoL via the EQ VAS form, a reliable and valid depiction of their QoL is obtained.

Assessment of prescription quality and medication appropriateness demands reliable tools. The medication appropriateness index (MAI) developed by Hanlon et al [17] has been shown to fulfil the criteria [17-19]. The MAI score is a reliable instrument to evaluate the elderly patient's drug therapy [20], to continuously question the treatment and the lack of follow up, to achieve better and more appropriate prescribing and most of all to minimize adverse drug events [3,21,22].

There are currently no studies that have definitively determined whether various methods designed to reduce drug-related problems in the elderly affect QoL [23]. The aim of our study was therefore to see if there is an association between medication quality and quality of life. We also wanted to examine if there is an association between medication quality and cognitive impairment.

## Methods

During the period September 2006 to May 2007, all patients ready for discharge from the University Hospital in Örebro, Sweden and fulfilling the criteria were eligible for the study. Inclusion criteria were: ≥ 75 years, ≥ 5 drugs and living in ordinary homes. Exclusion criteria were dementia, abuse (all forms of abuse registered in the patient's medication record) or malignant disease diagnosed before the study start. Moving to a nursing home during the study also resulted in exclusion. The electronic care planning system (Meddix), used throughout the County Council and municipalities, made the surveillance of all discharges complete and all patients had the same opportunity to be included. The study was performed in primary care, since the family physicians are responsible for the medical care of the elderly after discharge from hospital. The patients in the study were followed during one year with study end May 2008.

At time of discharge all patients were registered in the care planning system and a message was sent to the research centre. If the patient was eligible, a letter concerning the study including informed consent was sent to the patient.

**Figure 1:**
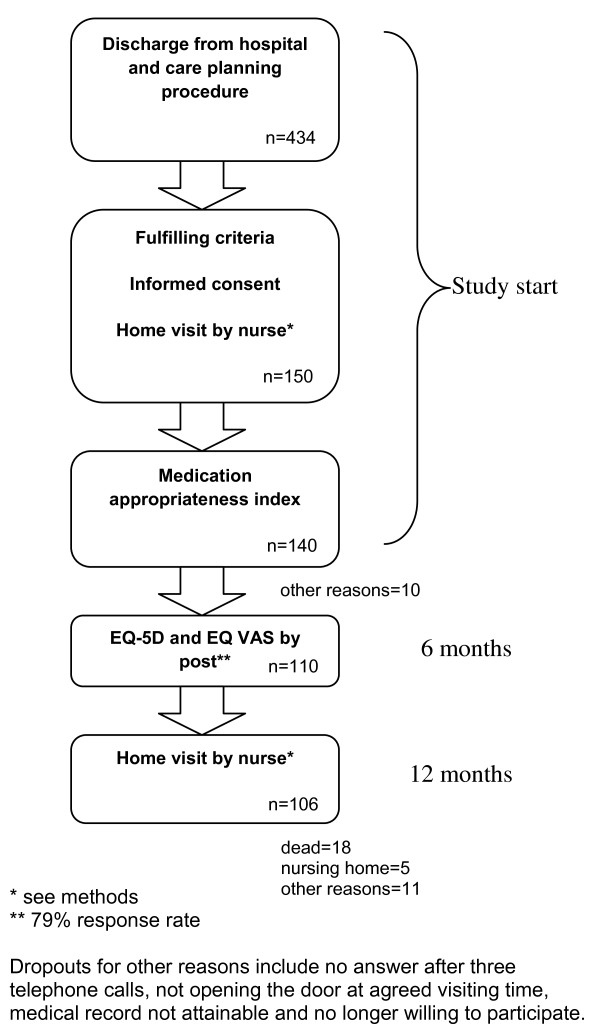
**Study flow chart**.

Within one month after discharge, a home visit was made (Figure 1). It consisted of questions about satisfaction and capability of managing the medication and the dosage regimen/dispensing and screening for cognitive impairment since this is often omitted and is a main issue for the patients' capability to handle their medication. Both the Mini Mental State Examination (MMSE) [24] and clock drawing test (CDT) were used, as the latter is more sensitive to decline in activities and orientation in daily life [25,26]. The patients also completed an EQ-5D and EQ VAS survey. The study nurse asked all patients about their drug regimen and compliance, to compare with their prescriptions. The "true" drug lists (the combinations of prescriptions from all physicians involved or previously involved in the patient's care) were then forwarded to the research centre. After six months all the patients received a letter with a new EQ-5D and EQ VAS survey. The study ended after 12 months with a follow-up home visit including EQ-5D, EQ VAS and questions of drug utilization. All the home visits throughout the study were done by the same study nurse.

To evaluate medication quality the MAI was used. This index has been developed by Professor Hanlon et al and was used after personal approval by Professor Hanlon. The MAI is considered to be the most reliable and valid comprehensive instrument of today [20]. It consists of explicit criteria and implicit judgment meaning it permits standardisation and takes advantage from clinical knowledge and judgment in the evaluation process [19,20]. The MAI review is based on thorough examinations of the patients' medication lists, prescriptions and medical records. Since all patients in the study had their medical care provided by the County Council, all data concerning the medical records and drug lists were available for the researchers. The medical record for every study patient was scrutinized systematically, by the same physician and research assistant throughout the study, according to the principles of MAI. Every drug was checked in accordance with the MAI routine on ten items regarding medication indication, effectiveness, dosage, directions, drug-drug interactions, drug-disease interactions, practicality, expense, duplication and duration [17,18]. This renders a weighted MAI score per drug ranging between 0 (good quality) and 18 (poor quality). In adherence with the principles of appropriate prescribing for elderly [3,21,27,28] the item of indication was deemed fundamental in our analysis and scoring of MAI. The assessment of indications was based on the patients' medical records.

Every patient's medical record was scrutinized systematically for each drug:

1. Was there an evident diagnose admitting prescription?

2. If not; were there any notes of a diagnose or symptom two years before, during or one year after the study?

3. If no diagnose was evident were there signs of ongoing follow-up of a specific disease, for example blood pressure or blood tests like lipids, thyroid hormone and glucose?

If any of these three conditions were fulfilled the drug was considered to have an indication. If the reviewed drug was determined to be devoid of indication, the grade C was given which in our analysis resulted in a C in all the nine following questions. Hence the drug received the worst (highest) possible MAI score. The total MAI score for each patient is calculated as the sum of the individual drug MAIs for that patient.

To measure QoL and functional status the validated questionnaire EQ-5D was used after approval of the EuroQol group. EQ-5D is a generic instrument evaluating function in five dimensions (mobility, self-care, usual activities, pain/discomfort and anxiety/depression) [16,29]. The EQ-5D index was used for an overall estimation of QoL. The preference weights and the calculation algorithm we used in this study were determined in the UK using data from the Measurement and Valuation of Health Survey [30]. EQ VAS was used for self-rating of current health-related QoL.

The study participants were divided into three equal size groups, A, B and C. The third of the patients with the lowest MAI score (measured at study start) and therefore the "best" medication quality was allocated to group A. Group B and C represented the thirds with the "middle/centre" respectively the "worst" medication quality. The groups were then compared with respect to EQ-5D index and EQ VAS at the three measuring points (study start, 6 months and 12 months) and MMSE/CDT at baseline.

The Regional Ethics Committee of Uppsala University approved the study.

### Statistical analyses

The study groups were analysed with respects to EQ-5D index and EQ VAS measured at study start, 6 months and 12 months. Jonckheere-Terpstra trend test across groups was performed. It tests the alternative hypothesis that the population medians are ordered in a particular direction (that is, if there is a dose-response relationship).

To be able to correct for number of drugs, sex and age as possible confounding factors, we created a linear multiple regression model with the EQ-5D index utility as response variable. The explanatory variables of primary interest were total MAI score, sex, age and number of medications. We also performed similar calculations with EQ VAS as the response variable.

To adjust for comorbidities we used the Charlson Comorbidity Index [31].

In addition we analysed the different MAI groups with respects to MMSE and CDT using the Jonckheere-Terpstra test.

The data were analyzed using the SPSS program, version 15.

## Results

150 patients were identified for inclusion in the study (Figure 1). Table 1 shows the characteristics of our study population. The proportion of patients satisfied with their drug therapy and patients' self-rated ability to handle their drug therapy is presented in Table 1. 84% of the patients in the study claimed to be satisfied with their drug therapy but only 56% felt able to handle their drug regimen. 79% of our patients preferred life quality over long life. Notable is the fact that 32% of the participants had MMSE < 25 as well as reductions in CDT score indicating possible cognitive impairment. The number of deaths during the 12 month study period in group A, B and C were 5 (11%), 7 (15%) respectively 6 (13%). 1, 4 respectively 2 of these patients died within the first 6 months.

**Table 1:** Characteristics of the study population

	*Total* *n = 140*	*Group A* *n = 47*	*Group B* *n = 47*	*Group C* *n = 46*
Age; mean	83.4 (5.0)	83.3 (4.5)	84.3 (5.4)	82.7 (5.0)

Sex; women (%)	62.1	66.0	53.2	67.4

men (%)	37.9	34.0	46.8	32.6

Mini Mental State Examination (MMSE); 1) median, 2) mean	1) 27 (23 - 28) 2) 25.6 (3.8)	1) 26 (23 - 28) 2) 25.2 (3.5)	1) 27 (23 - 29) 2) 25.3 (4.6)	1) 27 (24 - 29) 2) 26.2 (3.1)

Clock Drawing Test (CDT); 1) median, 2) mean	1) 2.0 (1.0 - 3.0) 2) 1.8 (0.9)	1) 2.0 (1.0 - 3.0) 2) 1.9 (0.9)	1) 2.0 (1.0 - 2.0) 2) 1.7 (0.9)	1) 2.0 (1.8 - 3.0) 2) 1.9 (1.0)

Are satisfied with drug therapy (%)	84.3	85.1	87.2	80.4

Feel able to handle drug therapy (%)	55.7	63.8	44.7	58.7

Prefer life quality before long life (%)	79.3	78.7	78.7	80.4

The values are presented as mean (± SD), median (IQR) or percentage.

The results from calculating MAI are presented in Table 2 as are the number of drugs per patient. In addition to wrong dosages, interaction/duration problems etc, the fact that a relatively large part of drug regiments lack indication causes surprisingly high total MAI scores. Extreme polypharmacy, defined as taking ≥ 10 drugs was common and persistent in all three groups (Table 2). Some drugs are considered to pose special risks for the elderly [23]. These are presented in Table 3 together with percent of patients taking the drug and percent of prescriptions lacking indication.

**Table 2:** Drug treatment and Medication Appropriateness Index

	*Study start*			
	
	*Total*	*Group A*	*Group B*	*Group C*
Number of drugs per patient; median	10.0	8.0	10.0	12.0

Number of drugs lacking indication per patient; median	3.0	1.0	3.0	6.0

Number of drugs lacking indication per patient; min - max	0 - 15	0 - 2	2 - 4	4 - 15

MAI score median	54.0	18.0	54.0	108.0

MAI score mean	61.3	16.0	51.3	117.7

MAI score min - max	0 - 270	0 - 36	36 - 72	72 - 270

**Table 3:** Special risk drugs

	*Percent taking the drug*	*Percent lacking indication*
Analgesics (light), ongoing	40.1	36.3

Analgesics (midrange), ongoing	7.5	50.0

Analgesics (strong), ongoing	9.5	47.1

Bulk/laxatives, ongoing	22.4	67.9

Benzodiazepines (short acting), total	10.2	82.4

Benzodiazepines (long acting), total	4.8	66.7

Sleeping tablets, total	44.2	88.1

NSAID, total	5.4	50.0

Neuroleptics, total	3.4	100.0

PPI, totalt	27.9	57.9

Digoxin, total	13.6	35.0

Loop diuretics, total	59.9	18.6

SSRI, total	19	70.4

Other anticholinergics*, total	21.8	70.4

NSAID - Non-Steroidal Anti-Inflammatory Drug

PPI - Proton-Pump Inhibitor

SSRI - Selective Serotonin Reuptake Inhibitor

*Amitriptyline, Clomipramine, Clemastine, Desloratadine, Hydroxyzine, Loratadine, Montelukast and Tolterodine

QoL, measured by EQ-5D, is presented as recommended by the EuroQol group [16] (Table 4).

**Table 4:** Frequency distribution (profile) of the EQ-5D descriptive system at baseline

	*Group A* *(n = 47)*	*Group B* *(n = 47)*	*Group C* *(n = 46)*
*Mobility*			

no problems (%)	13	6	13

some problems (%)	78	85	80

confined to bed (%)	9	9	7

*Self-Care*			

no problems (%)	69	61	60

some problems (%)	24	28	33

unable to (%)	7	11	7

*Usual Activities*			

no problems (%)	48	56	31

some problems (%)	35	20	33

unable to (%)	17	24	36

*Pain/Discomfort*			

none (%)	31	22	22

moderate (%)	54	54	58

extreme (%)	15	24	20

*Anxiety/Depression*			

none (%)	54	46	45

moderate (%)	39	46	53

extreme (%)	7	8	2

The internal loss of follow up was ≤ 3 in all groups.

The results from our statistical analysis are presented in Table 5 and 6. The Jonckheere-Terpstra test shows that a lower medication quality is associated with a lower quality of life. EQ-5D index was statistically significantly different (declining for each group) among the groups (p = 0.001 at study start, p = 0.001 at 6 months and p = 0.013 at 12 months) as was EQ VAS (p = 0.026 at study start, p = 0.003 at 6 months and p = 0.007 at 12 months).

**Table 5:** Medication appropriateness and quality of life

Group	*EQ-5D index* *at study start*			*EQ-5D index* *at 6 months*			*EQ-5D index* *at 12 months*		
	
	Mean	Median	n=	Mean	Median	n=	Mean	Median	n=
A (lowest MAI score)	0.58	0.73	47	0.59	0.69	34	0.57	0.73	33

B (medium MAI score)	0.51	0.66	44	0.50	0.60	32	0.43	0.62	32

C (highest MAI score)	0.33	0.39	46	0.32	0.41	32	0.37	0.37	34

	p = 0.001			p = 0.001			p = 0.013		

Statistical analyses were done using Jonckheere-Terpstra trend test.

A higher MAI score equals worse medication quality.

A higher EQ-5D index represents better quality of life (range 0 - 1, though negative values are possible and represents status "worse than death").

**Table 6:** Medication appropriateness and quality of life

Group	*EQ VAS* *at study start*			*EQ VAS* *at 6 months*			*EQ VAS* *at 12 months*		
	
	Mean	Median	n=	Mean	Median	n=	Mean	Median	n=
A (lowest MAI score)	55.8	50.0	47	61.0	60.0	33	63.2	60.0	32

B (medium MAI score)	51.2	50.0	43	51.7	50.0	32	51.0	50.0	32

C (highest MAI score)	46.2	50.0	46	45.2	50.0	29	51.7	50.0	34

	p = 0.026			p = 0.003			p = 0.007		

Statistical analyses were done using Jonckheere-Terpstra trend test.

A higher MAI score equals worse medication quality.

A higher EQ VAS represents better self-rated quality of life (range 0 - 100).

The same analysis was performed after dividing the study group into two age groups (above and below median; ≤ 83, ≥ 84 years) and male/female groups to adjust for age and sex. Even with these small groups the results remain statistically significant for EQ-5D for 9 out of 12 comparisons (4 groups, 3 different points in time) and the trend towards lower EQ-5D with lower medication quality still remains between the groups. For EQ VAS the results were statistically significant for 7 out of 12 comparisons. The same trend with declining EQ VAS with lower medication quality remains.

When we performed the linear regression with EQ-5D index as the response variable and MAI groups, age, sex and number of drugs as explanatory variables we basically found similar results. The difference in EQ-5D index between group A and group C was statistically significant at the first two points in time but not at the 12 month measuring point (p = 0.019 at study start, p = 0.011 at 6 months and p = 0.233 at 12 months). There was no statistically significant difference between the middle group and the group with the highest MAI score. When performing the linear regression with EQ-5D index as the response variable and MAI groups, age, sex and Charlson Comorbidity Index as explanatory variables we found that comorbidity did not affect EQ-5D index. The difference in EQ-5D between MAI group A and group C was remained statistically significant at the all three points in time (p = 0.001 at study start, p = 0.002 at 6 months and p = 0.033 at 12 months). There was no statistically significant difference between the middle group and the group with the highest MAI score.

For EQ VAS, there was a statistically significant difference between group A and C at the six and 12 month measuring points but not at baseline (p = 0.052 at study start, p = 0.009 at 6 months and p = 0.042 at 12 months). As with EQ-5D index, there was no statistically significant difference between the middle group and the group with the highest MAI score.

Number of drugs had a statistically significant impact on both EQ-5D index and EQ VAS at all points in time. Sex or age did not affect either EQ-5D index or EQ VAS.

We also analysed the different MAI groups with respects to MMSE and CDT using the Jonckheere-Terpstra test. In our study group we could not find any indication that cognitive impairment is associated with low medication quality.

## Discussion

The main result of our study demonstrates an association between medication quality and QoL. Through the study and by using reliable instruments, MAI together with EQ-5D and EQ VAS, we have been able to visualize the association between inappropriate medication and low QoL. We found a remarkable high number of patients with inappropriate medication. The findings are of importance for the individual as well as the healthcare system since the vulnerable group of elderly with chronic health problems and chronic drug treatment is growing.

We find it remarkable that more than four out of five patients in the study are satisfied with their drug therapy while only slightly more than half the patients feel able to handle their drug regimen and the calculation of MAI shows us that medication quality is overall poor. A possible reason for the low self-rated capability to handle drug regimens is the fact that almost one third of the participants had MMSE < 25 as well as reductions in CDT score, indicating cognitive impairment. A reason for patients claiming to be satisfied with their drug therapy while not being able to handle it could be trust in the "good doctor" and fear of damaging the doctor-patient relationship by voicing concerns about their drug therapy [32].

An important aspect is whether the MMSE and CDT results in our study indicate the ability of the patients to properly fill in the EQ-5D. According to previous research the EQ-5D is well suited for evaluating QoL in a population with cognitive impairment [33].

It is a well established truth that drug treatment and polypharmacy in the elderly are risk factors for adverse drug reactions, hospitalization and mortality [22,34,35]. These are factors known to affect QoL. In this study we set out to see if medication quality could also be associated to life quality. The reason for this is that we wanted to study quality of drug treatment from a patient perspective. With increasing number of elderly who faces the problems that come with old age, chronic medication and chronic diseases, the real challenge for the healthcare of tomorrow is both "to help people live longer and feel better" [15]. To achieve this, the healthcare professions need to adopt new outcomes, including QoL. By choosing QoL as an outcome instead of solely treatment goals per se we wanted to accomplish more of a patient focus and a movement towards shared decisions by empowerment of patient participation.

Polypharmacy is a giant challenge in many ways, but the objective of our study is appropriateness of the prescriptions in a wide perspective, meaning the burden of drug treatment for each patient. Appropriateness of medication is therefore the key word in every part of the discussion, because if appropriate and needed then the benefits of the medications are obvious for optimizing QoL. But as shown here, in many cases there is no indication for the treatment which is devastating throughout the system and especially for the patient. Indication as the basic principle for prescribing is learned by every medical student and is emphasized in the regulations for physicians and also in the reimbursement system for drug treatment. A finding is that there might have been an indication once, but no one has done a follow up, no one has adjusted the dose, no one has defined the time for treatment or the costs. The presences of interactions remain unnoticed. All these are important factors for the patients undergoing treatment as it affects their QoL. For some types of drugs this can seem as an issue of low significance (for example laxatives and vitamin pills) but the list of inappropriate drugs in our patient group also includes pain killers, sleeping pills and diuretics and in the worst cases anticoagulants and insulin. In every respect these results show lack of systematic work in the prescription process. The use of MAI with its explicit and implicit criteria gives an extensive and to some extent depressive perspective and shows the omission to fulfill the obligations connected to drug treatment.

To prescribe drugs is important in medical treatment and demonstrates initiative and action, but good and appropriate prescribing demands many considerations. It involves evaluation of symptoms, follow up of effect, adjustment of dose and monitoring over time as well as deprescribing when indicated [21,28,36]. Prescribing for elderly demands special knowledge and close monitoring [23]. This includes courage to deprescribe and the necessity of avoiding the prescribing cascade [37]. For the elderly patients who have multiple health problems, the risks increase as there are often many prescribers with different specializations involved, focusing on their area of specialization and with no one taking an overall responsibility regarding the patient [23].

The patient's QoL has historically been neglected since other outcomes are judged more important. Today there are guidelines for treatment of individual diseases, but there is a lack of guidelines and goals for treatment of the elderly with many diseases [38]. In the healthcare system there are now established incitements and rewards for following the guidelines for drug treatment (number of patients with recommended prescriptions) while considering the patient's quality of life is subordinate.

Some limitations should be acknowledged. In this study we have used one measure of QoL, the EQ-5D index. This is probably the most recognized instrument for measuring QoL and it is extensively used in international studies. It is nevertheless possible that a different result would be obtained with a different measure of QoL. The same pertain to our chosen measure of medication quality.

The MAI scoring system does not take into account that a patient might lack certain drugs that could be beneficiary to them, i.e. underprescription. The possible reduction in QoL and associated costs resulting from this underprescription is therefore not taken into account in this study.

Our study concentrates on the population of elderly with multiple medications and chronic diseases. Conclusions from this study can therefore not be used to generalize about other parts of the population/community. It is also a small study. More and bigger studies are needed to investigate the impact of poor medication quality in the general population and to confirm the results from this study.

In this study it was not possible to separate disease groups from one another since all patients in the study were multi-diseased and had medical conditions from several different disease groups. If we would have been able to separate the different disease groups, and adjust for these in the analysis, we believe that we might have found a stronger relationship between medication quality and QoL. We believe that it is a possibility that poor medication quality in certain disease groups has a bigger impact on QoL than others. Further studies are needed to evaluate if and how poor medication quality in different disease groups affect QoL.

The strength of our study is that it is performed in care as usual. Another strength is the fact that we are describing a group of people that will keep growing as the base of the population pyramid in the western world is contracting while the top is expanding. This means that measures to improve medication quality in the elderly in order to improve QoL will be a way to change a lot for lots of patients. The fact that we are using the patients' self stated medication lists as a basis for evaluating their prescriptions is both a strength and a weakness. By doing this, we are more likely to capture what medications the patient is actually taking but we are also subject to the patients' forgetfulness or possible unwillingness to share information.

When applying to the Hippocratic Oath, physicians are taught to do well and not to harm. The hierarchic structure of healthcare has undergone tremendous changes but the patient is still in a weak position despite the ongoing discussion of patient participation and empowerment. In a world of pharmacological possibilities the debate regarding prescribing ought to be as prominent as ever. Concerning the elderly patient there must be a crusade finding the breaking point were the intention to do "well" and not to harm means to deprescribe or refrain from prescribing based on shared decision with the patient to prioritize their QoL.

## Conclusion

Drug treatment in the elderly is a huge challenge for healthcare. Since drug quality is related to the patient's quality of life, there is immense reason to continuously evaluate every prescription and treatment. The evaluation and if possible deprescribing should be done as a process where both the patient and physician are involved.

## List of abbreviations

CDT: Clock drawing test; MAI: medication appropriateness index; Meddix: electronic care planning system; MMSE: Mini Mental State Evaluation; QoL: quality of life

## Competing interests

The authors declare that they have no competing interests.

## Authors' contributions

INO participated in the design of the study, the statistical analysis and the drafting of the manuscript. RR participated in the statistical analysis and the drafting of the manuscript. PE participated in the design of the study and the drafting of the manuscript. All authors read and approved the final manuscript.
